# Supplementary material for: MetaRibo-Seq measures translation in microbiomes
Source: Nat Commun. 2020 Jun 29;11:3268. doi: 10.1038/s41467-020-17081-z (PMC7324362; doi:10.1038/s41467-020-17081-z)
Supplement: Supplementary file 10 — Supplementary Data 7 [file 41467_2020_17081_MOESM10_ESM.zip › File2/Confidence_High_Taxonomy/188133_out.krona.html]

Javascript must be enabled to view this page.

members
magnitude
magnitudeUnassigned
count
unassigned
taxon
rank

188133\_out

2

2
2
superkingdom

976
phylum
1

200643
class
1

order
1
171549

815
1
family

genus
1
816

47678
species
1

SRS014683\_contig\_number\_contig-100\_10948.10948

1239
1
phylum

91061
class
1

1385
1
order

1378
1
genus

species
1

SRS049448\_contig\_number\_contig-100\_29870.29871
84135
